# Personalised reprogramming to prevent progressive pacemaker-related left ventricular dysfunction: A phase II randomised, controlled clinical trial

**DOI:** 10.1371/journal.pone.0259450

**Published:** 2021-12-13

**Authors:** Maria F. Paton, John Gierula, Judith E. Lowry, David A. Cairns, Kieran Bose Rosling, Charlotte A. Cole, Melanie McGinlay, Sam Straw, Rowena Byrom, Richard M. Cubbon, Mark T. Kearney, Klaus K. Witte

**Affiliations:** 1 Leeds Institute of Cardiovascular and Metabolic Medicine, Multidisciplinary Cardiovascular Research Centre, University of Leeds, Leeds, United Kingdom; 2 Leeds Teaching Hospitals NHS Trust, Leeds, United Kingdom; 3 Leeds Institute of Clinical Trials Research, University of Leeds, Leeds, United Kingdom; Kurume University School of Medicine, JAPAN

## Abstract

**Background:**

Pacemakers are widely utilised to treat bradycardia, but right ventricular (RV) pacing is associated with heightened risk of left ventricular (LV) systolic dysfunction and heart failure. We aimed to compare personalised pacemaker reprogramming to avoid RV pacing with usual care on echocardiographic and patient-orientated outcomes.

**Methods:**

A prospective phase II randomised, double-blind, parallel-group trial in 100 patients with a pacemaker implanted for indications other than third degree heart block for ≥2 years. Personalised pacemaker reprogramming was guided by a published protocol. Primary outcome was change in LV ejection fraction on echocardiography after 6 months. Secondary outcomes included LV remodeling, quality of life, and battery longevity.

**Results:**

Clinical and pacemaker variables were similar between groups. The mean age (SD) of participants was 76 (+/-9) years and 71% were male. Nine patients withdrew due to concurrent illness, leaving 91 patients in the intention-to-treat analysis. At 6 months, personalised programming compared to usual care, reduced RV pacing (-6.5±1.8% *versus* -0.21±1.7%; p<0.01), improved LV function (LV ejection fraction +3.09% [95% confidence interval (CI) 0.48 to 5.70%; p = 0.02]) and LV dimensions (LV end systolic volume indexed to body surface area -2.99mL/m^2^ [95% CI -5.69 to -0.29; p = 0.03]). Intervention also preserved battery longevity by approximately 5 months (+0.38 years [95% CI 0.14 to 0.62; p<0.01)) with no evidence of an effect on quality of life (+0.19, [95% CI -0.25 to 0.62; p = 0.402]).

**Conclusions:**

Personalised programming in patients with pacemakers for bradycardia can improve LV function and size, extend battery longevity, and is safe and acceptable to patients.

**Trial registration:**

ClinicalTrials.gov identifier: NCT03627585.

## Introduction

Pacemaker implantation is a common and safe procedure that can be lifesaving and is associated with a marked improvement in quality of life [1]. Over one million people worldwide are implanted with a pacemaker each year [2]. Cross-sectional and retrospective studies have suggested that the most frequent long-term cardiovascular co-morbidity associated with right ventricular (RV) pacemaker therapy is left ventricular systolic dysfunction (LVSD) or chronic heart failure (CHF). Whether due to a specific pacemaker-induced cardiomyopathy or not, CHF is associated with significant healthcare costs, [3] high morbidity and mortality, and is much more common in pacemaker recipients than in the general population, being found in >50% of those with a high proportion of pacemaker-induced ventricular heart beats [4].

RV pacing is associated with an immediate reduction in left ventricular (LV) function [5]. Observational studies of longer term clinical outcomes in pacemaker recipients are hampered by the confounding of high cardiovascular co-morbidity. Nevertheless, in response to the relationship between RV pacing and LVSD and heart failure events, pacemaker manufacturers have each developed largely automatic algorithms to avoid unnecessary RV pacing, but these are variably applied with reprogramming occurring in only 9% of all in-clinic follow-ups [6]. We have previously described in an observational cohort that careful individualised programming to limit RV pacing in people with RV pacemakers can successfully reduce pacing requirements, and leads to an improvement in LV function with no adverse effects on quality of life [7]. To our knowledge, no prospective randomised controlled trial has ever explored the effects of reducing RV pacing on LV function and quality of life in patients without third degree heart block in the era of RV pacing avoidance algorithms.

The aims of this trial were therefore to describe the effects of personalised reprogramming to limit ventricular pacing on echocardiographic and patient-orientated clinical outcomes in patients with avoidable RV pacing.

## Methods

Data that support the findings of this study contain potentially identifying patient information. As per the study sponsor regulations, they are therefore available upon reasonable request (governance-ethics@leeds.ac.uk).

### Trial design

This was a double-blind, randomised, controlled, parallel group, phase II study of personalised reprogramming to avoid unnecessary RV pacing versus usual care in patients with RV pacemakers implanted to treat bradycardia. The primary outcome of the study was change in LV ejection fraction (LVEF) between baseline and 6 months, with secondary outcomes of LV dimensions, quality of life (QoL), serum NT-pro-BNP concentrations and pacemaker generator longevity.

### Participants

**Fig 1:**
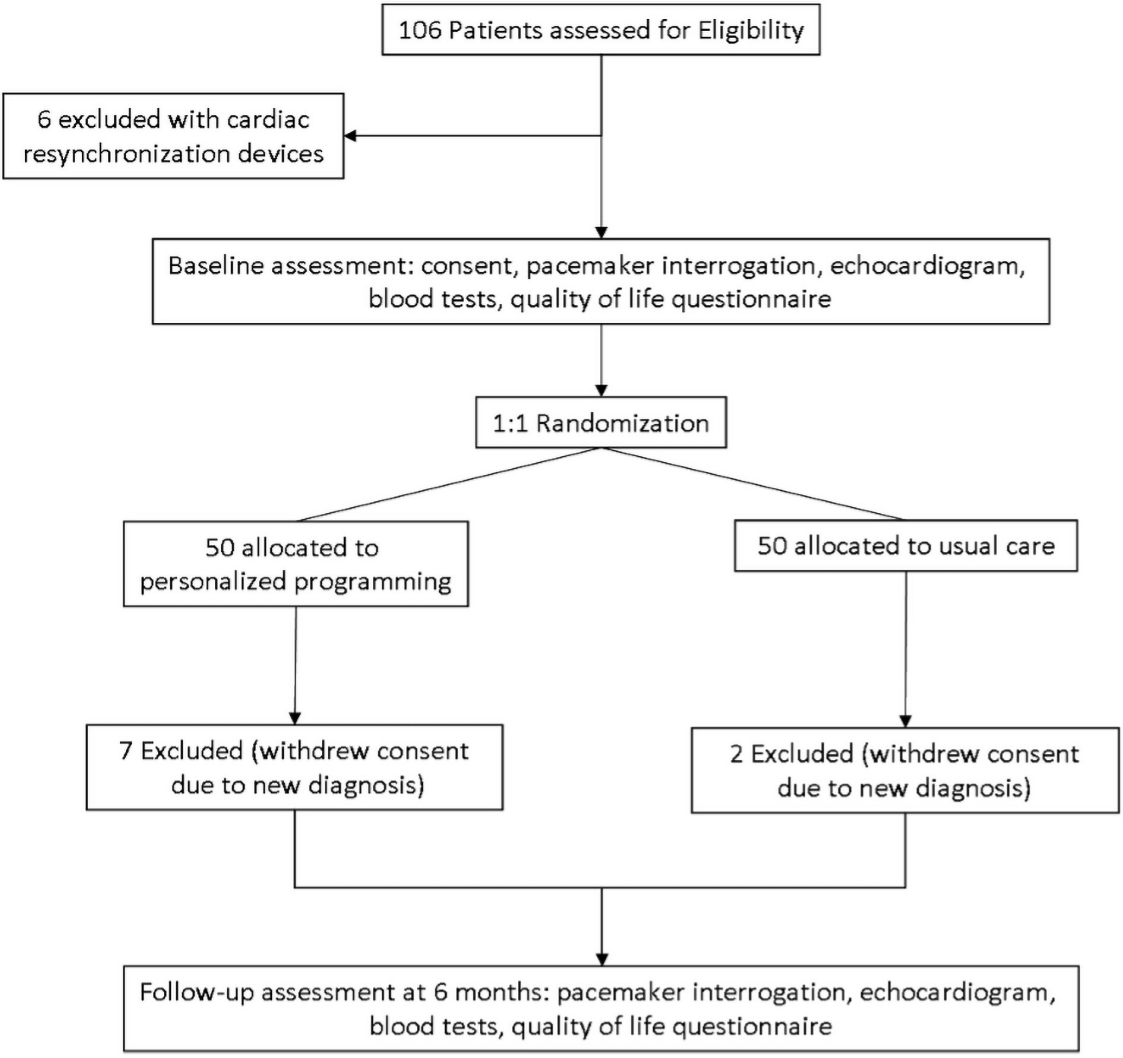
CONSORT participation flow diagram. Patient enrolment, randomisation, and disposition.

Unselected consecutive patients attending pacemaker follow-up clinics at Leeds Teaching Hospitals NHS Trust and Harrogate District Hospital NHS Foundation Trust were approached and invited to participate. To achieve a generalisable cohort representative of patients attending a pacemaker clinic, potential participants had to have had a RV pacemaker for at least two years, and we only included those able to provide written informed consent. We excluded people with third degree heart block, those with cardiac resynchronization therapy devices, and a pre-existing diagnosis of heart failure. Potential participants were offered a participant information sheet and contacted after one week. Those agreeing to participate attended for study visits at the NIHR Cardiovascular Clinical Research Facilities at either site and provided written informed consent (Fig 1).

### Study activities

During the baseline visit, information was collected on co-morbidities, past medical history, medication, pacemaker settings and a QoL assessment was undertaken using EuroQoL and a visual analogue scale of overall quality of life. A pacemaker interrogation was performed to document implant indication, percentage atrial and ventricular pacing, and programmed mode. We also undertook a transthoracic echocardiogram and a blood test to measure NT-pro-B-type natriuretic peptide (NT-pro-BNP).

### Echocardiographic techniques

Full two-dimensional transthoracic echocardiography was carried out with images recorded in two- and four-chamber views (GE Vivid E95, GE Healthcare, Milwaukee, Wisconsin). Images were stored in the EchoPAC digital imaging system (GE Healthcare) and analysed off-line. This analysis included a calculation of LV end-diastolic volume (LVEDV), LV end-systolic volume (LVESV) and LV ejection fraction (LVEF) using the biplane disks (modified Simpson) method by tracing the endocardial border, excluding the papillary muscles. The frame at the R-wave was taken as end-diastole, and the frame with the smallest LV cavity was considered to represent end-systole. The LVEDV and LVESV indexed to body surface area (LVEDVi and LVESVi) were calculated as LV volume/body surface area, where body surface area was calculated using the Mosteller equation [8]. To minimize observer bias, the analysis of cardiac ultrasound images of both studies was performed blinded to the images taken at baseline. These were measured by two experienced reviewers and inter-observer variability was assessed.

### Intervention

**Fig 2:**
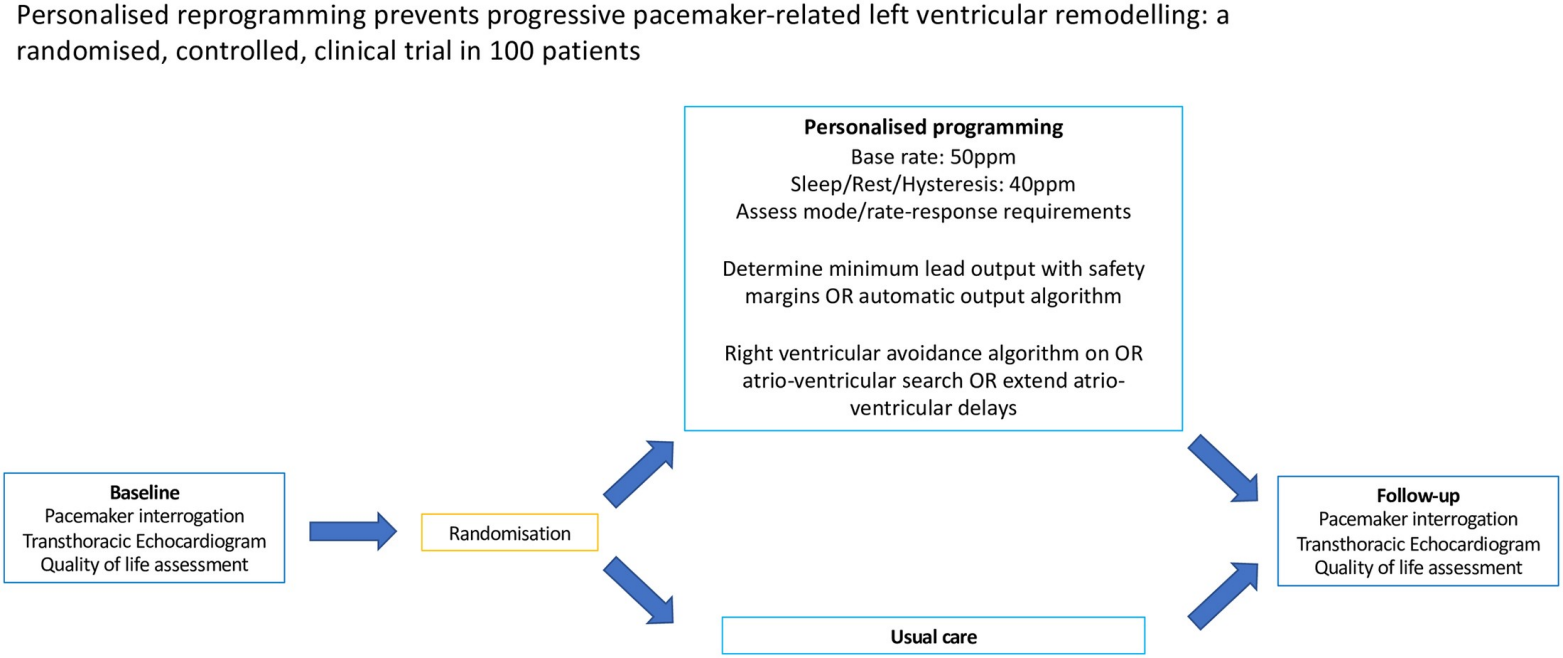
Pacemaker reprogramming protocol and its results. Simple adjustments to pacemaker programming in addition to manufacturer-specific algorithms can reduce RV pacing, improve LV function and dimensions, lengthen battery longevity with no loss to patient quality of life.

Following baseline testing, patients were allocated into two groups corresponding to either ‘usual care’ or ‘personalised reprogramming’. Usual care followed the National Institute for Health and Care Excellence (NICE) guidelines [9] and British Heart Rhythm Society standards [10] which include advice on the activation of RV pacing avoidance algorithms. In addition to following these guidelines, those allocated to the intervention arm underwent supplementary reprogramming to further promote intrinsic rhythm according to our previously published RV pacing avoidance algorithm. This included reducing day-time base rate (BR) to 50 beats per minute(bpm), and nocturnal or sleep rate (or hysteresis where available) to 40 bpm, deactivating rate-adaptive pacing, extending atrio-ventricular timing delays and reducing lead outputs (Fig 2).

### Follow-up

Patients were contacted at one week and one month by the unblinded team member to ensure short term acceptability and safety, and were then reviewed at six months when all tests (echocardiogram, pacemaker check, quality of life assessment, and NT-pro-BNP blood test) were repeated.

### Outcomes

Our primary outcome was change in LVEF between baseline and 6 months post-randomization. Key secondary outcomes, assessed simultaneously, included remodeling variables (LVEDVi, LVESVi), QoL (EQ5D-3L), and remaining estimated battery longevity.

### Sample size

Our sample size was influenced by our pilot data [7]. We estimated that allocating at least 70 patients, in a 1:1 ratio to the usual care and reprogramming arms (35 in each group) would allow us to describe a 95% confidence interval for a clinically relevant difference in mean LVEF of 5% at 6 months post-randomization with a power of 80% [11]. Due to interest from patients approached, recruitment was increased to 100 participants prior to study unblinding taking place.

#### Randomisation and concealment

Participants were allocated to usual care or reprogramming using random number generation, stratified by centre. If allocated to intervention, their device was programmed according to our previously published algorithm [7] by an unblinded team member not involved with recruitment, data collection or analysis. The patient and the investigator (MFP), who worked across both sites for consistency, were blinded to allocation throughout the course of the study.

### Statistical analyses

Analyses followed a predefined plan as set out in the trial protocol (NCT: 03627585). Normality for all continuous variables was tested using the Shapiro-Wilk test. Normally distributed continuous variables were reported as mean and standard deviation (SD), and non-normally distributed continuous variables as median and 25^th^-75^th^ percentiles. Categorical variables were presented as count and percentages. Differences between interventional groups’ baseline characteristics were assessed using the 2-sample Student’s t-test for normally distributed data and the Mann-Whitney U test for non-normally distributed data, whilst categorical variables were compared using the χ^2^ test. Analysis of covariance (ANCOVA) was conducted to assess inter-group significant differences for outcome variables controlling for baseline values, with Bonferroni correction to adjust for multiplicity. All across-treatment group comparisons were two-sided and are presented as mean change with 95% confidence intervals (95% CI). A p-value <0.05 was deemed statistically significant for comparisons.

Reproducibility of echocardiographic measures between two blinded echocardiographic readers (MFP and CAC) were described by intraclass correlation coefficient (ICC). Data were analysed using the Statistical Package for the Social Sciences SPSS version 26 (IBM Corp., Armonk, New York), R: A Language and Environment for Statistical Computing version 3.2.3 (R Development Core Team, Vienna, Austria), and SAS version 9.4 (SAS Institute, Inc., Cary, North Carolina).

### Patient and public involvement

The research question was driven by patients with pacemakers presenting to local HF clinics with symptoms suggestive of CHF, a deterioration in cardiac function and a high burden of RV pacing. The study was initially discussed with a well-established local patient and public involvement advisory group (PPI-AG) consisting of people with cardiac devices, cardiovascular disease and their families. The PPI-AG advised on suitable follow-up periods, study procedures, information sheets and dissemination plans. They were particularly interested to know the potential effect on battery longevity of the intervention, as their primary concern was the number of generator replacements required over a lifetime, particularly for an increasingly frail population.

### Funding and ethical considerations

Funding for the trial was through an NIHR Doctoral Fellowship award (MFP). Following ethical review by East Midlands Research Ethics Committee, the trial was approved by the Health Research Authority of the United Kingdom (16/EM/0337) and was registered on ClinicalTrials.gov (NCT:03627585). Full registration was retrospective due to an error in the submission process. However the authors confirm that all ongoing and related trials for this drug/intervention are registered.

## Results

A total of 100 patients were recruited from two centres between January 2017 and September 2018. Of the 100 patients, 9 withdrew due to serious illness, all of which were non-cardiovascular. No patients reported changes to their medical therapy during the study period. No patient randomised to intervention reported adverse effects from device reprogramming (Fig 1).

Patient and clinical characteristics were similar between intervention groups for age, sex, baseline RV pacing burden, LVEF, NT-proBNP and pacemaker battery longevity (Table 1). More than half (71%) were male with a mean age of 76 (SD±9) years. Co-morbidities included diabetes mellitus (31%), history of myocardial infarction (13%), percutaneous coronary intervention (10%), previous coronary artery bypass grafting (8%). Patients had a median atrial pacing burden of 27 (3–67)% and RV pacing percentage of 9 (1–58)% with a mean resting heart rate of 69 (±12)bpm. Mean LVEF was 50 (±9), with no evidence of a difference in those with a baseline LVEF ≥50% (preserved LVEF) and without (reduced LVEF) between interventional groups (27 randomised to personalised programming vs 32 to usual care; p = 0.31), and median NT-proBNP was 1423 (±3783)pg/mL.

**Table 1:** Patient characteristics at baseline.

	Total	Interventional Group		p-value
		Personalised Programming	Usual Care	
	(n = 100)	(n = 50)	(n = 50)	
**Patient Demographics**				
**Age (years)**	76 (9)	75 (10)	76 (9)	0.579
**Sex (male)**	71 (71)	35 (70)	36 (72)	0.368
**Height (cm)**	169 (15)	170 (10)	167 (19)	0.376
**Weight (kg)**	82 (19)	84 (19)	80 (20)	
**Atrial Rhythm**				
Atrial Fibrillation	39 (39)	22 (44)	17 (34)	0.450
Paced	3 (3)	2 (4)	1 (2)	
Sinus Rhythm	58 (58)	26 (52)	32 (64)	
**Clinical History Data**				
**Myocardial Infarction**	13 (13)	6 (12)	7 (14)	0.766
**Diabetes Mellitus**	31 (31)	20 (40)	11 (22)	0.052
**CABG**	8 (8)	3 (6)	5 (10)	0.461
**PCI**	10 (10)	4 (8)	6 (12)	0.505
**CVA**	18 (18)	9 (18)	9 (18%)	0.962
**NYHA**				
**I**	48 (48)	27 (54)	21 (42)	0.230
**II**	52 (52)	23 (46)	29 (58)	
**Haemodynamic Data**				
**Resting Heart Rate (bpm)**	69 (12)	69 (12)	69 (12)	0.150
**Resting Systolic BP(mmHg)**	138 (23)	138 (22)	138 (24)	0.158
**NT-pro-BNP (pg/ml)**	1423 (3783)	1368 (3028)	1473 (4396)	0.894
**Pacing Data**				
**Pacing indication**				
Atrioventricular block	30 (30)	15 (30)	15 (30)	0.602
Sinus Node Disease	69 (69)	34 (68)	35 (70)	
Other	1 (1)	1 (2)	0 (0)	
**Dual chamber pacemaker**	91(91)	45(90)	46(92)	0.500
**Time since first pacemaker (years)**	11 (7)	12 (8)	11 (5)	0.317
**Atrial pacing proportion (%)**	27 (3–67)	25 (4–69)	31 (3–68)	0.796
**Ventricular pacing proportion (%)**	10 (1–58)	9 (1–73)	11 (2–42)	0.841
**Echocardiographic Data**				
**LVEF (%)**	50 (9)	49 (10)	50 (9)	0.732
**Preserved EF (>/= 50%)**	59 (59)	27 (54)	32 (64)	0.310
**LVEDV (mL)**	107 (78–122)	106 (66–122)	107 (77–124)	0.802
**LVESV (mL)**	47 (37–60)	47 (37–60)	44 (37–60)	0.924
**LVESVi (mL/m** ^2^ **)**	24 (15–31)	24 (20–31)	23 (20–33)	0.710
**LV diastolic dysfunction grade**				
**Normal**	20 (20)	47 (14)	13 (26)	0.368
**I**	50 (50)	27 (54)	23 (46)	
**II**	29 (29)	15 (30)	14 (28)	
**III**	1 (1)	1 (1)	0 (0)	
**Medical Therapy Data**				
**Beta-blocker**	45 (45)	19 (38)	26 (52)	0.159
**ACEi/ARB**	47 (47)	23 (46)	24 (48)	0.841
**Furosemide**	21 (21)	10 (20)	11 (22)	0.806

Continuous normally distributed data are expressed as mean (SD), non-normally distributed continuous data as median (IQR) or categorical data as n (%).

CABG; coronary artery bypass grafting, PCI; percutaneous coronary intervention, CVA; cerebrovascular attack, BP; blood pressure, LVEF; left ventricular ejection fraction, LVEDV; left ventricular end diastolic volume, LVESV; left ventricular end-systolic volume, LVESVi; left ventricular end-systolic volume index, ACEi; Angiotensin-converting-enzyme inhibitor, ARB; Angiotensin II receptor blocker.

Distribution of device manufacturers is shown in (S1 Table). There were no significant differences in pacemaker lead site or type between the groups. All patients were paced at the right ventricular apex. Approximately half of the participants had a passive atrial lead (52% in the usual care group vs 50% randomised to personalised programming; p = 0.50), and 82% had a passive ventricular lead (82% in the usual care group vs 83% randomised to personalised programming; p = 0.54).

Patients were followed for a median of 189 days (interquartile range 176, 230), similar between intervention groups. No patient developed third degree AV block and no patient experienced an acute coronary syndrome or new or worsening heart failure event requiring hospitalisation or urgent ambulatory visit during follow-up. At baseline, most patients were programmed in DDD or VVI mode with rate response active in 40%, and an average base rate of 54(±6)bpm (S2 Table). Despite usual care already including pacing avoidance algorithms, supplementary personalised pacemaker programming successfully achieved an additional reduction in RV pacing percentage compared with usual care (-7% [95% confidence interval -11 to -2%; p = 0.01]). This was achieved by a reduction in base rate (in 44%), reductions in sleep, rest or hysteresis rates (68%), altered mode (8%), deactivating rate-adaptive pacing mode(38%), activating atrioventricular search algorithms (4%), activating automatic threshold algorithms (36%), reducing lead outputs (74%) and deactivating electrocardiogram storage where not necessary (10%). These proportions were similar across manufacturers. During the follow-up period, there were no changes to guideline directed standard of care programming in those allocated usual care. Changes in the pacemaker programming at follow-up between the groups are outlined in (S2 Table). There was no evidence of a statistical or clinically significant difference in incidence of atrial fibrillation during the 6 month follow-up in patients randomised to personalised programming compared to usual care (-3±16% vs. -2±16%; p = 0.66).

### Primary outcome measure

**Fig 3:**
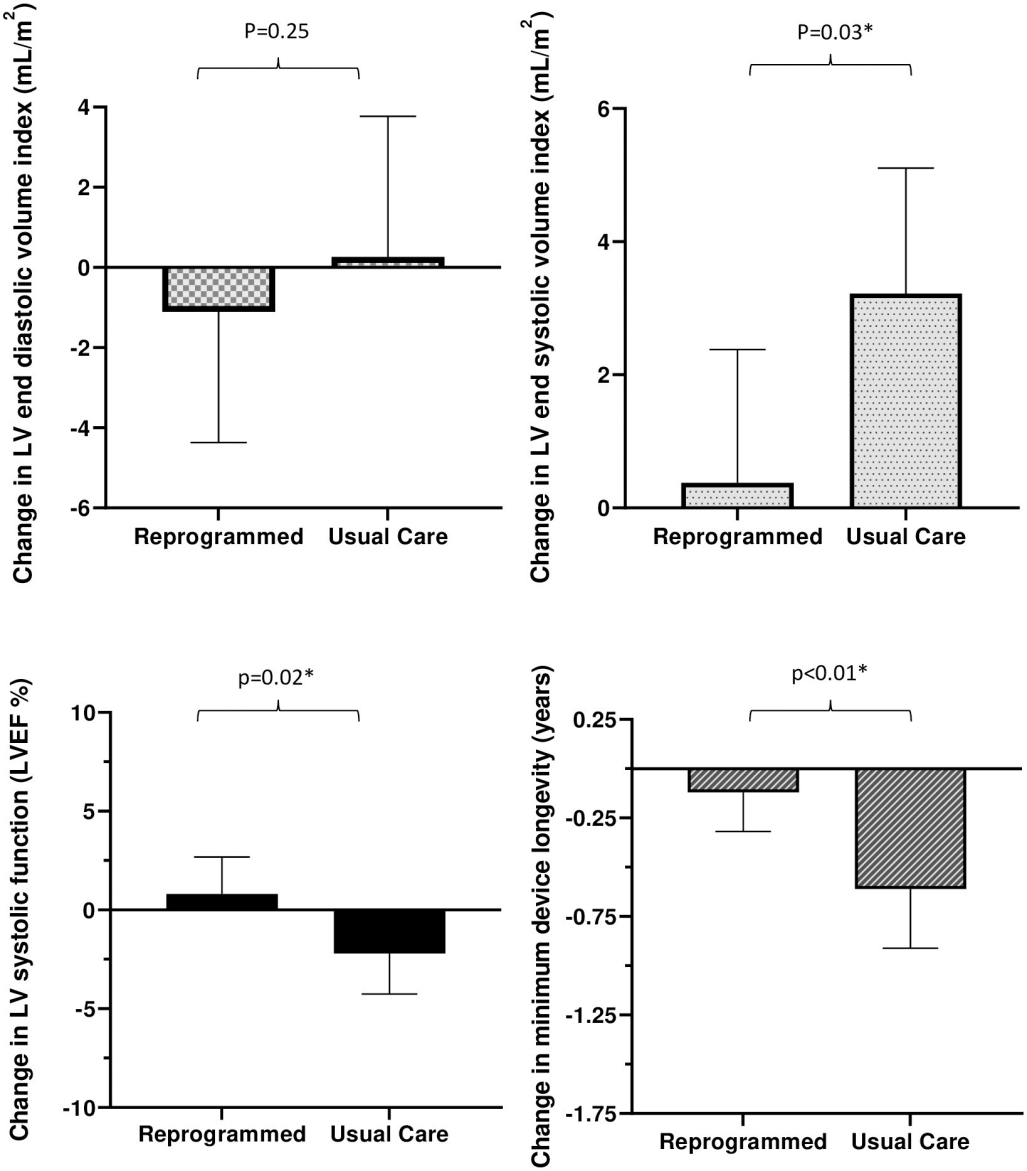
Primary and secondary outcomes by allocation. Mean change and standard deviation in LV diastolic and systolic volume index, left ventricular ejection fraction and minimum device longevity following 6 months of usual care programming versus personalised programming.

There was an improvement in the primary endpoint of LV systolic function, measured by LVEF, at 6 months in patients randomised to receive personalised pacemaker programming when compared with those receiving usual care (mean difference (+3.09% [0.48 to 5.70%; p = 0.02]) (Fig 3 and Table 2).

**Table 2:** Change in primary and secondary outcome variables in patients following 6 months of personalised pacemaker programming v usual care: Intention-to-treat analysis.

Outcome	Randomised treatment	Mean at follow-up [95% Confidence Interval]	Mean difference [95% Confidence Interval	P value
**Primary outcome**				
LVEF (%)	Reprogramming	51.05 [49.15, 52.94]	**+3.09 [0.48, 5.70]**	**0.02** *
	Usual care	47.96 [46.16, 49.75]		
**Secondary outcomes**				
LVEDV (mL)	Reprogramming	104.30 [96.99, 111.61]	-4.81 [-14.72, 5.11]	0.34
	Usual care	109.10 [102.41, 115.80]		
LVESV (mL)	Reprogramming	52.99 [49.20, 56.78]	-5.08 [-10.26, 0.11]	0.06
	Usual care	58.07 [54.44, 61.60]		
LVEDVi (mL/ m^2^)	Reprogramming	53.33 [49.72, 56.97]	-2.88 [-7.83, 2.07]	0.25
	Usual care	56.22 [52.85, 59.59]		
LVESVi (mL/m^2^)	Reprogramming	26.95 [24.99, 28.90]	**-2.99 [-5.69, -0.29]**	**0.03** *
	Usual care	29.93 [28.08, 31.79]		
NT-proBNP (pg/mL)	Reprogramming	1136.22 [768.02, 1504.43]	-87.83 [-450.95, 626.61]	0.75
	Usual care	1224.05 [831.08, 1617.02]		
Battery Longevity (years)	Reprogramming	6.15 [5.98, 6.32]	**+0.38 [0.14, 0.62]**	**<0.01** *
	Usual care	5.77 [5.60, 5.93]		
EQ5D	Reprogramming	0.69 [0.38, 1.02]	+0.19 [-0.25, 0.62]	0.40
	Usual care	0.51 [0.22, 0.81		
EQ-VAS	Reprogramming	74.44 [68.97, 79.90]	-0.03 [-7.60, 7.54]	0.99
	Usual care	74.47 [69.25, 79.69]		

Values are mean change [95% confidence intervals]; 95% significance shown in bold,

*Denotes significance (P<0.05).

LVEF; left ventricular ejection fraction, LVEDV; left ventricular end-diastolic volume, LVESV; left ventricular end systolic volume, LVESVi; left ventricular end systolic volume index, NT-proBNP; N-terminal pro-B-type natriuretic peptide, EQ5D; Euro-quality of life score -5 questions, VAS; visual analogue scale.

### Secondary outcome measures

There was a reduction in mean LVEDVi of -2.88mL/m^2^ (95% CI -7.83 to 2.07; p = 0.249) mL/m^2^ in those allocated to personalised care, compared to those receiving usual care, when corrected for baseline LVEDVi, although our sample did not provide sufficient statistical evidence to conclude this effect exists. Moreover, patients randomised to personalised programming had a significantly smaller LVESVi at follow-up than those randomised to usual care (-2.99mL/m^2^ [95% CI -5.69 to -0.29 mL/m^2^; p = 0.03]) (Fig 3 and Table 2). Of those allocated personalised programming, 8 (30%) experienced a clinically significant reduction in LVESVi by greater than 15% compared to 2 (7%) patients randomised to usual care (p = 0.02). We did not see a difference in atrial fibrillation burden during follow-up.

Personalising pacemaker settings to avoid unnecessary RV pacing had no detrimental effect on quality of life as measured by EQ5D-3L (+0.19 [95% CI -0.25 to 0.62; p = 0.402]) and visual analogue scale (VAS) (-0.03 [-7.60 to 7.54; p = 0.99]) when adjusted for baseline quality of life.

Furthermore, personalised programming led to an extension of remaining minimum battery longevity by approximately 5 months compared with usual care (+0.38 years [0.14 to 0.62 years; p<0.01]) (Fig 3 and Table 2).

### Subgroup analysis

An exploratory subgroup analysis showed in those with a preserved LVEF at baseline, LVEF improved by a mean of +4% (95%CI 0.94 to 7.57; p = 0.01) with personalised programming. In participants with a reduced LVEF (<50%) at baseline, there was a non-significant mean increase in LVEF of 2% (95%CI -2.03 to +6.71%; p = 0.29) in those allocated personalised programming.”

Analysis of the primary outcome in the first 70 recruited patients shows an improvement in LVEF with personalised programming which did not meet statistical significance (+2.52% [95%CI -0.44 to 5.49%; p = 0.09]) (S3 Table).

### Reproducibility of echocardiographic measurements

Echocardiographic outcome measurements demonstrated strong inter-observer agreement for LVEF [ICC 0.968 (0.948 to 0.980)], LVEDV [ICC 0.955 (0.931 to 0.975)], and LVESV (ICC 0.964 (0.941 to 0.979)].

## Discussion

The present study is the first to provide evidence that even when right ventricular pacing burden is already low, and pacing avoidance algorithms are already activated, personalising pacemaker programming to limit RV pacing even further leads to a clinically relevant improvement in LV systolic function and prevents further remodeling, with no evidence of an effect on quality of life, whilst simultaneously preserving battery longevity.

### RV pacing-associated LV dysfunction

RV pacing has a longstanding association with an acute reduction in LV contractility. In two separate cross-sectional studies of unselected patients with pacemakers, we have described and validated that the degree of LV dysfunction is strongly related to the amount of RV pacing, and that this relationship is enhanced by the presence of cardiovascular disease [5, 6]. Hence, most patients with LV dysfunction attending a pacemaker clinic will not fulfill the exacting criteria of pacemaker-*induced* cardiomyopathy [4]. rather the RV pacing will be a *contributor*.

Longer term effects on LV function are reported to include myocardial perfusion and structural abnormalities [12]. thought eventually to contribute to the initiation and progression of LV adverse remodeling with the subsequent progression to LVSD and CHF. However, whether this LV dysfunction is progressive, and the underlying mechanisms behind the heterogeneity in functional response to RV pacing are both currently unknown.

### LV remodeling in RV pacemaker patients

Reverse remodeling is increasingly appreciated as a surrogate for improved patient-orientated outcomes, due to a close relationship between therapy-related changes in echocardiographic variables (in HF patients) and subsequent findings in morbidity and mortality studies of the same interventions. A pooled analysis of interventional clinical trial data has shown that every 5% absolute increase in mean LVEF is associated with an odds ratio of 0.86 (95% CI 0.77 to 0.96) for mortality [13]. Similar data apply to LV volumes. A reduction in LVESVi of ≥15% is associated with better outcomes in recipients of cardiac resynchronisation therapy (CRT), [14] and has been used as an endpoint in CRT studies previously. Our data suggest therefore, that a long-term application of personalised pacemaker therapy could improve outcomes.

### Does RV pacing-associated LV impairment affect outcomes?

Observational data in pacemaker recipients without clinical heart failure at baseline has also shown higher heart failure-associated deaths and hospitalisation rates as RV pacing burden increases, the risk of which is highest within the first 6 months post-pacemaker implantation [15, 16]. Although these studies could not prove causation, the most consistent feature predicting mortality in each of these studies was cardiac dysfunction at baseline and a high RV pacing proportion.

### Can RV pacing be avoided?

In experimental models, reducing RV pacing seems to correct *pacing-induced* left ventricular systolic dysfunction. We previously undertook an observational study in 66 patients with long term pacemakers to determine whether reducing RV pacing in a chronically implanted patient cohort had effects upon LV function [7]. On an intention-to-treat analysis, the protocol reduced mean RV pacing percentage by 49% (95% CI 41 to 57%; p<0.0001) from baseline, and there was an improvement in LVEF of 6% (95% CI 4 to 8%; p<0.0001) and a reduction in LV dimensions.

Since our initial work, manufacturers’ bespoke RV pacing avoidance algorithms have become standard of care, hence the present study was carried out on a background of low RV pacing burden in patients not necessarily fulfilling the precise diagnosis of pacemaker-induced cardiomyopathy. The impact of RV pacing on cardiac function is likely to be variable in the context of contributing factors such as co-morbidities, but there may be a dose-response relationship over a lifetime of pacemaker therapy. This study is the first to confirm that simple pacing adjustments are well tolerated and also that even a modest further reduction in RV pacing burden is associated with improvements in LV size and function, hence, any attempt to reduce pacing burden is a worthwhile endeavor.

Exploratory subgroup analysis also showed that personalising pacemaker programming is as important to those with a preserved LVEF. Patients with preserved LVEF at baseline experienced a larger absolute increase in LVEF. One could hypothesise that potentially the earlier in the patients disease status we implement personalised programming, the more likely we are to preserve LV size and function, but certainly personalised programming seems to be beneficial in all patients. As the present study is a phase II study, a larger investigation with greater power is required to extend these findings into clinical endpoints of relevance to patients.”

### The opportunity to improve device longevity

Whilst avoiding RV pacing and improving or maintaining LV function is likely to be beneficial, device longevity is the most important aspect of pacemaker therapy to patients and has featured in the medical and the lay press [17]. The amount of pacing the device has to perform is the major drain on battery current and studies have previously suggested reprogramming can have beneficial effects on predicted pacemaker longevities [18] and therefore potentially reduce the complication rate of patient undergoing generator replacement procedures.

The results of this study therefore provide the first data from a randomised, placebo-controlled trial of chronically implanted pacemakers, that even many years following implant, and despite widespread use and promotion of RV pacing avoidance technology by companies and guidelines, careful programming using our algorithm further reduced RV pacing and had other positive effects, such as a preservation of battery longevity. Addressing programming earlier in the life of a pacemaker battery is likely to have cumulative effects upon device longevity.

### Safety and patient tolerability

Our protocol, which included a lowering of the base rate and deactivating rate-adaptive pacing, was well-tolerated with no patients returning with or reporting symptoms and no detriment to their quality of life. This finding is consistent with previous data where rate-adaptive pacing has been associated with highly variable effects on measures of quality of life [19].

### International and national guidelines

Currently international guidelines state that RV pacing should be avoided if possible, [9, 10, 20] but they offer limited advice on how this should be done. Clinical practice often relies on local policy and how pacemakers are programmed is highly variable. The present data describe benefits on clinical outcomes and battery longevity which, if included in guidelines, have the potential to not only reduce clinical event rates but also improve generator longevity—both key drivers of cost effectiveness.

## Limitations

Although this trial was performed within two centres, they reside within a single region in the UK, potentially limiting generalisability, particularly as international pacemaker programming may differ. However, baseline demographic data indicate that our population was representative of a pacemaker recipient population.

We did not limit enrolment only to patients with a pacing-induced cardiomyopathy, defined by a specific reduction in LVEF from pre-implant levels due to the presence of the pacemaker [3, 4]. We chose instead to enroll unselected patients attending a routine pacemaker clinic, with a view to exploring the benefits across the population in whom the pacemaker is likely to be contributing, rather than the primary cause of LV dysfunction. Our data suggest that even in the absence of a proven pacemaker-induced cardiomyopathy, optimised programming should be undertaken.

Estimated battery longevity remaining, rather than actual battery longevity, was utilised as a pragmatic measure of longevity due to the variable data available at follow-up between manufacturers. Whilst energy consumption or battery voltage may have provided additional information, this was not routinely obtainable from all devices included in the study, nor is it routinely available clinically.

Although the present study demonstrated modest benefits on LV function and battery longevity from personalised programming, these are likely to be cumulative, especially if initiated early following implantation. Longer term follow-up in a larger multi-centre trial including patients with a greater severity of sinus node disease would allow for further sub-analysis to understand which programming modifications show the greatest effect.

## Conclusions

Personalised reprogramming to avoid unnecessary RV pacing in unselected patients with RV pacemakers, beyond manufacturers’ pacing avoidance algorithms, can improve LV function, extend battery longevity, and is safe and acceptable to patients.

## Supporting information

S1 Table Device manufacturer distribution. (DOCX)

S2 Table Pacemaker programming at baseline and follow-up. (DOCX)

S3 Table Change in primary outcome in the first 70 patients recruited following 6 months of personalised pacemaker programming v usual care: Intention-to-treat analysis. (DOCX)

S1 Checklist CONSORT 2010 checklist of information to include when reporting a randomised trial*. (DOC)

S1 Protocol (DOCX)
