# Supplementary material for: Personalised reprogramming to prevent progressive pacemaker-related left ventricular dysfunction: A phase II randomised, controlled clinical trial
Source: PLoS One. 2021 Dec 13;16(12):e0259450. doi: 10.1371/journal.pone.0259450 (PMC8668131; doi:10.1371/journal.pone.0259450)
Supplement: S1 Table — (DOCX) [file pone.0259450.s001.docx]

| **S1 Table: Device Manufacturer Distribution** | | | | | | |
| --- | --- | --- | --- | --- | --- | --- |
|  | **Total** | | **Interventional Group** | | | |
|  |  |  | **Personalised Programming** | | **Usual Care** | |
|  | **(n=100)** | | **(n=50)** | | **(n=50)** | |
| **Device Manufacturer** | |  | |  | |  |
| **Medtronic** | 59 (59) | | 32 (64) | | 27 (54) | |
| **Abbott** | 21 (21) | | 10 (20) | | 11 (22) | |
| **Boston Scientific** | 19 (19) | | 8 (16) | | 11 (22) | |
| **Vitatron** | 1 (1) | | 0 (0) | | 1 (2) | |
