# Supplementary material for: Personalised reprogramming to prevent progressive pacemaker-related left ventricular dysfunction: A phase II randomised, controlled clinical trial
Source: PLoS One. 2021 Dec 13;16(12):e0259450. doi: 10.1371/journal.pone.0259450 (PMC8668131; doi:10.1371/journal.pone.0259450)
Supplement: S2 Table — (DOCX) [file pone.0259450.s002.docx]

| **S2 Table: Pacemaker Programming at Baseline and Follow-up** | | | |
| --- | --- | --- | --- |
|  | **Baseline** | **Follow-Up** | |
|  | **Usual Care Programming** | **Personalised Programming** | **Usual Care Programming** |
|  | **(n=100)** | **(n=43)** | **(n=48)** |
| **Base rate (ppm)** | 54 (±6) | 50 (±3) | 54 (±6) |
| **Atrial Energy Consumption (μW)** | 3.8 (±6.4) | 2.9 (±4) | 3.3 (±2.7) |
| **Ventricular Energy Consumption (μW)** | 4.0 (±3.5) | 3.5 (±4.1) | 3.8 (±4.1) |
| **Paced AV Delay (ms)** | 199 (±55) | 203 (±54) | 195 (±56) |
| **Sensed AV delay (ms)** | 171 (±60) | 179 (±53) | 157 (±65) |
| **Rate Response Active** | 40 (40) | 10 (23) | 21 (44) |
| **Sleep +/- Hysteresis Active** | 41 (41) | 37 (86) | 15 (31) |
| **Mode**  AAI+  DDD  DDI  VVI  VDD | 31 (31)  28 (28)  4 (4)  35 (35)  2 (2) | 14 (28)  10 (20)  2 (4)  16 (32)  1 (2) | 15 (30)  16 (32)  1 (2)  15 (30)  1 (2) |
| Continuous normally distributed data are expressed as mean (SD) or categorical data as n (%). | | | |
