# Supplementary material for: Personalised reprogramming to prevent progressive pacemaker-related left ventricular dysfunction: A phase II randomised, controlled clinical trial
Source: PLoS One. 2021 Dec 13;16(12):e0259450. doi: 10.1371/journal.pone.0259450 (PMC8668131; doi:10.1371/journal.pone.0259450)
Supplement: S3 Table — (DOCX) [file pone.0259450.s003.docx]

| **S3 Table: Change in primary outcome in the first 70 patients recruited following 6 months of personalised pacemaker programming v usual care: intention-to-treat analysis** | | | | |
| --- | --- | --- | --- | --- |
| **Outcome** | **Randomised treatment** | **Mean at follow-up**  **[95% Confidence Interval]** | **Mean difference**  **[95% Confidence Interval** | **P value** |
| **Primary outcome** | | | | |
| LVEF (%) | Reprogramming | 53.09 [50.98, 55.20] | +2.52 [-0.44, 5.49] | 0.09 |
|  | Usual care | 50.56 [48.48, 52.64] |  |  |
|  | Usual care | 74.47 [69.25, 79.69] |  |  |
| Values are mean change [95% confidence intervals]; 95% significance shown in bold, *Denotes significance (P<0.05).  LVEF; left ventricular ejection fraction, LVEDV; left ventricular end-diastolic volume, LVESV; left ventricular end systolic volume, LVESVi; left ventricular end systolic volume index, NT-proBNP; N-terminal pro-B-type natriuretic peptide, EQ5D; Euro-quality of life score -5 questions, VAS; visual analogue scale. | | | | |
