# Supplementary material for: Personalised reprogramming to prevent progressive pacemaker-related left ventricular dysfunction: A phase II randomised, controlled clinical trial
Source: PLoS One. 2021 Dec 13;16(12):e0259450. doi: 10.1371/journal.pone.0259450 (PMC8668131; doi:10.1371/journal.pone.0259450)
Supplement: S1 Protocol — (DOCX) [file pone.0259450.s005.docx]

**Title:** Reprogramming to Prevent Progressive Pacemaker-induced Remodelling

**Summary:**Left ventricular (LV) systolic dysfunction (LVSD) is common (~40%) in patients with long term right ventricular (RV) pacemakers. It is thought to be due to the long term effects of dyssynchrony (disturbed timing) induced by RV pacing on LV contraction which leads to adverse remodelling.

**Hypothesis:**Pacing-related left ventricular remodelling can be safely limited or reversed through personalised programming while also extending battery longevity.

**Aims:**The aim of the current proposal is to demonstrate that pacemaker-associated adverse LV remodeling can be prevented or reversed by careful pacemaker programming.

**Background**

Pacemaker implantation is a common and safe procedure that can be lifesaving and is associated with a marked improvement in quality of life. Around 350,000 people in the UK have a pacemaker and 40,000 new pacemakers are implanted annually.[1] Cross-sectional and retrospective studies have suggested that the most frequent long-term complication associated with RV pacemaker therapy is LVSD or chronic heart failure (CHF). Although CHF is common in the general population,[2] expensive of resources,[3][4][5] and associated with a high morbidity and mortality, CHF in pacemaker clinic is much more common than in the general population, can be found in >50% of those with a high proportion of RV paced beats,[6][7] and is associated with a 10% annual hospitalisation rate.[6]

*Underlying pathophysiology of right ventricular pacing and its effects on left ventricular function*

Acute RV pacing is associated with a reduction in LV contractility,[8[9][10] but also has detrimental effects on diastolic function.[10][11][12][13] Longer term effects on LV function might also include the consequence of increased myocardial catecholamine activity,[14] and abnormal myocardial perfusion.[14][15][16][17] In combination, these effects lead to structural [18] and histological abnormalities [19] and abnormal gene expression [20] eventually contributing to the development of LVSD and heart failure. In experimental models, reducing RV pacing seems to correct LVSD.[15][17]

*The relationship between new RV pacing and heart failure events*

There are only a few studies describing the clinical effects of RV pacing in a real-world situation. Early studies demonstrated that patients paced in the RV had a higher rate of subsequent congestive heart failure (HF) than those paced mainly in the right atrium,[21] and that HF events were especially frequent in patients requiring long-term pacemakers for congenital complete heart block.[22] These studies have been supported by more recent work showing that patients with sick sinus syndrome paced in the right atrium have fewer HF events, fewer strokes and less atrial fibrillation than those paced only in the RV.[23] The MOST study demonstrated a threshold of 40% RV pacing in dual chamber (atrium and ventricular) mode (DDD) and 80% in ventricular pacing (VVI) above which there were 2.6 and 2.5-fold increases in HF hospitalization rate following pacemaker implantation.[24] However, this study was designed to look at the development of atrial fibrillation; no echocardiograms were done at baseline or at follow-up and other risk factors for HF were not described. One other study suggested a prevalence of heart failure events of 26% at almost 8 years in people receiving new pacemakers: older patients, those with electrical dyssynchrony and a history of ischaemic heart disease were at higher risk.[25] In contrast, in the largest study so far, in 1415 patients with new pacemakers for sick sinus syndrome, the amount of ventricular pacing did not influence the development of HF.[26] Crucially, these studies all present the change in LV function following pacemaker implantation. None describe changes in patients with existing devices.

*RV pacing and left ventricular remodeling*

Few longitudinal studies have described the adverse remodeling effects of RV pacing, and patients with existing LVSD are frequently excluded, limiting the real-world applicability. Furthermore, all of these have focussed on newly implanted patients describing the changes in the first months following the implant. For example in the study by Nielsen examining regional blood flow in response to RV pacing,[15] the 15 patients randomized to DDD pacing experienced a reduction in LVEF (SE) from 61 (0.09) to 56 (0.07)% at one year. In another study, there was a reduction in LVEF from 55 (3) to 47 (3)% after 18 months in the 12 patients allocated RV apical pacing.[17] Finally, a study of 166 patients exposed to either high (≥90% VP) or low (mean 17% VP) rates of RV pacing showed a difference in change in LV end-systolic volumes 32 (18) to 43 (48), v 32 (18) to 35 (19)mm and LVEF 61 (8) to 56 (10) v 60 (8) to 57 (9) after 2.9 years.[27]

In summary, no study has ever demonstrated whether changes in LV function or remodeling are progressive in long-term pacemaker patients and which factors (for example pacing-induced dyssynchrony, or previous cardiovascular conditions) contribute to exacerbate the effects of RV pacing on LV function. Knowledge of whether or not there is an ***ongoing*** pathophysiological process has important implications for the timing and overall benefit of applying any reprogramming protocol in patients with long-term pacemakers.

*Predicting pacing-associated LV dysfunction*

In two separate cross-sectional studies we have described and validated that the degree of LV function is strongly related to the amount of RV pacing, and that this relationship is enhanced by the presence of cardiovascular disease (Figure 1).[6][7]  We have also determined that the presence of LVSD is the most powerful determinant of medium-term survival.[6] Whether remodeling is ***progressive*** in patients with long-term pacemakers is unknown.

Furthermore, the effect of pacing-induced dyssynchrony measured on echocardiography is also unclear. One study in 91 patients with RV pacemakers following atrio-ventricular node ablation demonstrated that dyssynchrony (as measured by apex to base mechanical propagation delay) was associated with a >5% reduction in LVEF at 28 months.[28] However, no absolute changes in the entire group were described. In contrast, in 19 patients the mean reduction of LVEF from 65% to 56% following one year of RV pacing was not predictable based upon dyssynchrony measures, although the change in LVEF was greater in those with >60% ventricular pacing.[29] Once again these are studies in patients with new implants. There are no data describing whether dyssynchrony, however measured, contributes to progressive remodeling in patients with existing pacemakers.

*Does RV pacing-induced LV remodeling affect outcomes?*

In patients with LVSD, RV pacing is associated with adverse outcomes.[30] Retrospective [31][32][33][34][35][36] and prospective [25][37] studies of pacemakers in patients without heart failure, have looked at death or hospitalization but none have tied this to pacing-related changes in cardiac function. Nevertheless, it is notable that the most consistent feature in each of these studies was that cardiac dysfunction at baseline and complete heart block as an indication were important markers of mortality,[25][31] although age, coronary artery disease, co-morbidities (chronic airways disease and diabetes mellitus), paced QRS,[38] and atrial fibrillation were also relevant.[39]

*The unrecognised relevance of battery longevity and how it might be improved by reprogramming*

Our patient advisors and the PPI-AG have confirmed previously published data that battery longevity is the most important aspect of pacemaker therapy to them.[40] My experience and that of my colleagues is that remaining battery longevity is the first question we are asked by patients during pacemaker follow-up appointments. The issue of battery longevity has recently been raised in the medical and the lay press.[41] Our data suggest that extending longevity by as little as twelve months could avoid a pacemaker generator replacement procedure entirely in around 20% of patients (and therefore eliminate its 1-5% complication rate [42]).

Finite battery longevity is partly due to the environment in which the devices work. Reliably creating a uniform electrical pulse using a simple chemical reaction within a sealed unit is challenging. The products of this reaction cannot be vented as in normal batteries, and eventually hinder the reaction itself making electricity generation unreliable. Hence merely increasing battery size, although acceptable to patients,[43] might not be the most appropriate (or most straightforward) approach. On the other hand, since the amount of pacing the device has to perform is the major drain on battery current, our protocol of limiting RV pacing to protect LV function might lead to a secondary benefit of an extension of battery longevity.

Reducing the output on the pacing leads is effective in extending longevity,[44][45], but although one observational study has described the benefit of careful programming to improve battery longevity,[46] and another the benefit of an automatic device-specific program to do this,[47] optimisation remains consistently underutilised in daily practice. Our pilot data in patients scheduled for generator replacement (see below) demonstrated an improvement in battery longevity by a mean of 6 months (range 3-36 months). It is likely that addressing programming earlier in the life of a pacemaker battery will have cumulative effects upon the longevity of the device. A secondary aim of my project therefore is to provide (the first) pilot data from a randomised, placebo-controlled trial on the effects of our programming protocol on estimated battery longevity.

***Underpinning pilot data:****Can RV-pacing be avoided and what effect does this have?*

We undertook an observational study to determine whether a high proportion of RV paced beats is associated with LVSD and also to explore the effects of reducing RV pacing in a chronically implanted patient cohort.

Methods

The study involved 66 patients with long-term pacemakers.[48] All subjects underwent baseline echocardiography, pacemaker check, exercise test (if capable), and quality of life assessment. We followed a prespecified protocol as outlined in Figure 3, reducing day-time base rate (BR) to 50 beats per minute (and nocturnal or sleep rate (or hysteresis where available) to 40 beats/minute) and deactivated rate-adaptive pacing. In patients with SR and heart block we extended atrio-ventricular (AV) timing delays to avoid RV pacing, and in those with particularly long AV delays at the time of battery replacement implanted a device that offered a program capable of avoiding RV pacing such as the managed ventricular pacing (MVP) algorithm by Medtronic. Patients were reassessed at six months with the same tests as at baseline.

The primary endpoint was the change in LVEF from baseline to six months. Echocardiographic images were anonymised and analysed in random fashion by a single blinded observer.  LVEF was calculated as the mean for three non-paced, normally conducted beats using the modified Simpson’s rule (bi-plane). We powered our study based upon an estimated improvement in LVEF of 5% of on a baseline LV ejection fraction of 45% (SD 12) in patients requiring >50% ventricular pacing based upon previous studies of CRT and our own pilot data.[49]

Results

Our programming changes were tolerated in all but two patients. Both of these patients had atrial fibrillation and were reprogrammed back to their original setting of a BR of 60 beats per minute with rate-adaptive pacing re-activated after one week with no long term adverse effects. These patients were included in the analysis. All patients returned for their 6-month reassessment. At follow-up mean [95% CI] resting heart rates were lower by 8 [6-10] beats per minute (p<0.0001), with no difference between patients in AF and SR. Our strategy reduced mean RV pacing percentage by 49 [41-57]%, (p<0.0001) from baseline and was greater in patients with SR than in those with AF (-61 [49-73] v -40 [30-50]%; p=0.01). Although there was an additional reduction in %VP with the use of pacing avoidance algorithms (-67 [52-80] v -53 [28-76], this was not statistically significant.

Reducing the percentage of heart beats stimulated by the pacemaker through programming was associated with an improvement in LVEF of 6 [95% CI 4-8]%; p<0.0001 from baseline and a reduction in LV dimensions. The ΔVP% with our intervention was greater in patients with SR, but there was no discordance in the response for LVEF by atrial rhythm (or by strategy), although patients with AF had a greater magnitude of improvement in LVEDD (p<0.02 between SR and AF). There was a relationship between ΔVP% and change in LVEF (r=0.26; p=0.04)(Figure 4): a reduction in RV pacing was associated with an improvement in LVEF,

There was no overall change in NT-pro-BNP levels or exercise variables (including peak heart rates) in the cohort as a whole or when divided by atrial rhythm although there was a trend towards a relationship between ΔVP% and change in NT-pro-BNP levels (r=0.27; p=0.07). This relationship was significant in patients with AF (r=0.44; p=0.03). Quality of life (by EuroQoL EQ5D) was not influenced by reductions in pacing percentage (including deactivating rate-adaptive pacing).

**Plan of investigation**

Design: This will be a randomised, double-blind interventional study of personalised reprogramming to avoid RV pacing versus standard care.

Recruitment: We will enrol sequential patients agreeing to be involved with one of our other studies exploring the relationship between pacemakers and heart function who have avoidable RV pacing.

Population:

*Inclusion criteria*

1. Chronic RV pacemaker implant (at least 24 months)
2. Willing and able to give informed consent for the intervention

*Exclusion criteria*

1. Known poor imaging quality (details of patients excluded for this reason will be recorded)
2. Patients with complete heart block with no reprogramming options

Methods:

We will invite patients attending for the assessment in our observational studies to participate. Each patient will be offered an information sheet for the present protocol. We will contact them at one week and if interested, they will come back to the NIHR Clinical Research Facility at Leeds General Infirmary or the Cardiology Department at Harrogate District Hospital.

All patients will then be randomised using a simple random number generator to either standard care or personalised reprogramming according to a predetermined protocol (appendix 3). Randomisation and reprogramming will be undertaken by a colleague. The patient and the investigator will remain blinded to allocation. Patients will be contacted at one week and one month by the same unblinded cardiac physiologist to ensure short term acceptability and safety, and will then be reviewed at six months when all tests will be repeated.

At the follow-up visit at 6 months, the tests (echocardiogram, pacemaker check, quality of life assessment, and BNP blood test) will be repeated.

*Image recording and image analysis*

Full conventional echocardiography will be carried out with grey-scale and tissue Doppler images recorded in the two and four chamber views using harmonics to improve border definition if necessary. All images will be stored in the ‘echopac’ digital imaging system and analysed offline. I will therefore be blinded to the clinical status of the patient. This analysis will include a calculation of LV end diastolic and end systolic volumes using the real-time three dimensional data and two-dimensional biplane discs (modified Simpson’s) method by tracing the endocardial border excluding the papillary muscles. An average of three measurements will be used in the final analysis. The frame at the R-wave will be taken as end diastole, and the frame with the smallest LV cavity, as end systole. The LV end-systolic volume index (LVESVi) will be calculated as LVESV/body surface area.

For two-dimensional measures of dyssynchrony, narrow sector angle acquisitions of each LV wall will be recorded in apical 4-. 2- and 3-chamber views with a minimum frame rate of 90Hz. Time to isometric contraction, maximum sustained systolic velocity, minimum strain and early and late diastolic velocities will be measured in 12 non-apical segments and dyssynchrony indexes will be calculated as the standard deviation from each of the 12 segments.[50] We have stored TDi images for all patients who came to the initial assessment.

Real-time three-dimensional (RT3D) echocardiographic images aiming for full volume acquisition of the entire left ventricle from the apex to the atrio-ventricular valves will also be recorded at the follow-up visit during breathhold. This dataset can then be used to carefully measure LV volumes, dimensions and thereby ejection fraction using a semi-automated endocardial border detection process. This has greater reproducibility for cardiac function and dimensions than two-dimensional datasets.[51] The LV cavity is recreated mathematically which also provides time-volume information for the entire cardiac cycle and can be broken up to estimate time-volume data for each of the 16 standard myocardial segments. The standard deviation of the time to reach minimal regional volume for these segments is the systolic dyssynchrony index (SDI),[51][52] expressed as a percentage of the cardiac cycle rather than absolute time to correct for cycle length. Much of this is automated in current machines. The SDI is a reliable predictor of response to cardiac resynchronisation therapy,[52] but has not yet been used to examine dyssynchrony and its relationship to remodelling in patients with standard pacemakers. The reproducibitlity of RT3D dyssynchrony assessment is reported as (mean, SD) 0.47+/-1.43%. Simple techniques to standardise approach can improve reproducibility results.[53]

To avoid observer bias, the analysis of the follow-up cardiac ultrasound images will be performed blinded to the images taken at baseline. However, to gain a complete dataset that includes the dyssynchrony variables which were recorded but not analysed, I will also reanalyse images from patients’ first visits. I will report 10% of the scans twice in order to describe intra-observer reproducibility. These will be randomly chosen during the study and will also be reported by a colleague to describe interobserver reproducibility.

these follow-up images will be analysed blinded to the data from patients’ first attendance using RT3D echocardiography to improve reproducibility. RT3D echocardiography has better Intra-operator reproducibility for LVEF and LV end-diastolic volume than standard two-dimensional images at (mean +/- SD) 1.75 (2.99)% for ejection fraction and 3.6 +/- 11.9mL respectively.[52] Inter and intra-observer reproducibility will be described as outlined above.

*Statistical considerations*

Our sample size has been influenced by our pilot data which revealed a 5% (SD) improvement on a background mean of 43 [95% CI 39-45]%. I will therefore recruit 35 patients to each arm of this study.

This is a single-centre phase II trial and therefore we estimate that randomising 70 patients 1:1 to the intervention and control arms will allow us to estimate a 95% confidence interval for the difference in mean LVEF at 6m post-randomisation of width +/-5. This allows for the drop-out of 6 patients (~10%). Furthermore, although this trial is not powered primarily to show a significant difference, this number of patients still allows 50% power at a 5% significance level to detect a difference of 6 percentage points in mean LVEF post-randomisation from 45% in the control arm to 51% in the intervention arm.

*Statistical analysis plan*

Our primary endpoint will be LVEF at 6m post-randomisation and secondary outcomes will include other remodeling parameters (LVEDV, LVESV), quality of life (EQ5D-5L), and estimated battery longevity at 6m post-randomisation. Patients will be analysed according to their randomised allocation and the difference between groups estimated using a general linear model adjusting for baseline LVEF. Analysis of other endpoints will use identical methodology.

*Choice of endpoints*

Left ventricular remodelling is an accepted surrogate endpoint in clinical studies.[59] A recent review has linked therapy-related changes in the three main echocardiographic variables (in HF patients) to the subsequent findings in morbidity/mortality studies of the same interventions.[60] Overall, an intervention that led to a  5% increase in mean LVEF was associated with an odds ratio (OR) of 0.86 [95%CI 0.77 – 0.96] for one-year mortality or a 4.9 fold increase in the odds of surviving one year  [95%CI 1.2-20.3] in subsequent mortality trials. LV end diastolic volume (LVEDV) also demonstrated a reliable link to mortality outcomes. A decrease in 10ml was associated with an OR of 0.95 for mortality at one year [CI 0.94-0.97] and a 1.9-fold [1.2-3.2] increase in the odds that an intervention would show a favourable outcome. A decrease in LVESV of 10ml was associated with a relative OR of 0.96 [0.93-0.98] for mortality. In one study of cardiac resynchronization therapy (CRT), a reduction in LVESV of >10% had a sensitivity and specificity of >70% each for all-cause mortality and 87 and 69% for cardiovascular mortality.[61] These relationships are not seen with changes in 6-minute walk distance datasets, or for changes in peak oxygen consumption or natriuretic peptide levels.[62]

We will also measure change in LVESVi which is frequently used in pacemaker studies.[54] A reduction in LVESVi of ≥15% is associated with better outcome in recipients of a CRT device,[49] and this cut-off has been used as an endpoint in one large study of cardiac resynchronization therapy versus standard pacing for heart block.[54]

Remaining battery longevity is modelled upon usage and lead variables to the time of the pacemaker check. I will document the estimated remaining longevity at the first and second visits and compare the changes in longevity estimates between groups.

*Safety and patient tolerability*

Our protocol of de-activating rate-adaptive pacing was well-tolerated by both groups of patients in our observational study. There was no reduction in peak heart rate or peak oxygen consumption and no difference in quality of life. Rate-adaptive pacing in pacemaker patients without CHF is associated with a greater cardiac output rise during exercise,[64] and although studies have shown improvements in exercise capacity ranging from the marginal,[65][66] to the dramatic,[67] there is no consistency on measures of quality of life in either AF or SR.[68][69][70] Our recent data have described a weak relationship between heart rate rise and exercise capacity in patients with LVSD, and no benefit from rate adaptive pacing on exercise capacity.[71] Hence, the pilot data that have led to this proposal are consistent with the literature, that rate-adaptive pacing is of little benefit in most patients with standard pacemakers and especially those with LVSD. In contrast however, as we have shown, de-activating rate-adaptive pacing can lead to significant reductions in ventricular pacing.

**Justification of importance and health, health care and services to patients and public**

Pacemaker implantation is one of the most commonly performed procedures in the NHS and, with an ageing population, implant rate continues to increase nationally. The procedure is safe and reliable but RV pacing can lead to LVSD and HF, thereby increasing mortality and morbidity. Although potential alternatives to RV pacing have been proposed, each increase the complexity of the procedure, limiting access for patients and increasing cost and complication rates, without clear benefits over the standard approach.

This project will deliver a double-blind randomised placebo-controlled study that will demonstrate the safety, acceptability, and possible efficacy on LV function and battery preservation effect of careful pacemaker programming to avoid RV pacing. This personalized approach with appropriate patient stratification and tailored programming will improve the cost-effectiveness of pacemaker therapy, provide data to allow the targeting of newer pacing strategies to those most likely to gain benefit and is therefore of crucial importance to patients, their carers, and the wider NHS as a whole.

References

. http://www.ucl.ac.uk/nicor/audits/cardiacrhythm/about

2. Cowie MR, Mosterd A, Wood DA. The epidemiology of heart failure. *Eur Heart Journal* 1997;**18:**208-25

3. Sutton GC. Epidemiologic aspects of heart failure. *Am Heart J* 1990;**120:**1538-40

4. McMurray J, Dargie HJ. Trends in hospitalisation for chronic heart failure in the United Kingdom. *Eur Heart J* 1992;**13**(suppl):350

5. McMurray J, McDonough T, Morrison CE, Dargie HJ. The growing problem of heart failure in Scottish hospitals. *Br Heart J* 1993;**69**(suppl):73

**6. Gierula J, Cubbon RM, Jamil HA, Byrom RJ, Waldron ZL, Pavitt S, Kearney MT, Witte KK. Patients with long-term permanent pacemakers have a high prevalence of left ventricular dysfunction. *J Cardiovasc Med* 2015;16:743-50**

**7. Thackray SD, Witte KK, Nikitin NP, Clark AL, Kaye GC, Cleland JG. The prevalence of heart failure and asymptomatic left ventricular systolic dysfunction in a typical regional pacemaker population. *Eur Heart J* 2003;24:1143-52**

8. Koch E. Der Kontraktionsablauf an der Kammer des Froschherzens und die Form der entsprechenden Suspensionkurve, mit besonderen Ausfu¨hrungen u¨ber das Alles-oderNichts-Gesetz, die Extrasystole und den Herzalternans. *Pflügers Arch Physiol* 1920;**181:**106–29

9. Wiggers CJ. The muscular reactions of the mammalian ventricles to artificial surface stimuli. *Am J Physiol* 1925;**73:** 346–78

10. Heyndrickx G, Vilaine J, Knight D, Vatner S. Effects of altered site of electrical activation on myocardial performance during inotropic stimulation. *Circulation* 1985;**71:**1010–6

1. Betocchi S, Piscione F, Villari B, et al. Effects of induced asynchrony on left ventricular diastolic function in patients with coronary artery disease. *J Am Coll Cardiol* 1993;**21:**1124–31

2. Bedotto J, Grayburn P, Black W, et al. Alterations in left ventricular relaxation during atrioventricular pacing in humans. *J Am Coll Cardiol* 1990;**15:**658–64

3. Stojnic B, Stojanov P, Angelkov L, Pavlovic S, Radjen G, Velimirovic D. Evaluation of asynchronous left ventricular relaxation by doppler echocardiography during ventricular pacing with AV synchrony (VDD): Comparison with atrial pacing (AAI). *PACE* 1996;**19:**940–4

4. Lee MA, Dae MW, Langberg JJ, Griffin JC, Chin MC, Finkbeiner WE, O’Connell JW et al. et al. Effects of long-term right ventricular apical pacing on left ventricular perfusion, innervation, function and histology. *J Am Coll Cardiol* 1994;**24:**225–32

5. Nielsen JC, Bøttcher M, Nielsen TT, Pedersen AK, Andersen HR. Regional myocardial blood flow in patients with sick sinus syndrome randomized to long-term single chamber atrial or dual chamber pacing--effect of pacing mode and rate. *J Am Coll Cardiol* 2000;**35:**1453-61

6. Tse HF, Lau CP. Long term effect of right ventricular pacing on myocardial perfusion and function. *J Am Coll Cardiol* 1997;**29:**744-49]

7. Tse HF, Yu C, Wong KK, Tsang V, Leung YL, Ho WY, Lau CP. Functiopnal abnormalities in patients with permanent right ventricular pacing: the effects of sites of electrical stimulation. *J Am Coll Cardiol* 2002;**40:**1451-58

8. van Oosterhout MF, Prinzen FW, Arts T, Schreuder JJ, Vanagt WY, Cleutjens JP, Reneman RS. Asynchronous electrical activation induces asymmetrical hypertrophy of the left ventricular wall. *Circulation* 1998;**98:**588-95

9. Adomian G, Beazell J. Myofibrillar disarray produced in normal hearts by chronic electrical pacing. *Am Heart J* 1986;**112:**79–83

20. Arkolaki EG, Simantirakis EN, Kontaraki JE, Chrysostomakis SI, Patrianakos AP, Chlouverakis GI, Nakou ES, Vardas PE. Alterations in the expression of genes related to contractile function and hypertrophy of the left ventricle in chronically paced patients from the right ventricular apex. *Europace* 2015;**17:**1563-70

21. Rosenqvisl M. Brandt J. Scholler H. Long-term pacing in sinus node disease: effects of stimulation mode on cardiovascular morbidity and mortalily. *Am Heart J* 1988;**116:**16-22

22. Thambo JB, Bordachar P, Garrigue S, Lafitte S, Sanders P, Reuter S, Girardot R, Crepin D, Reant P, Roudaut R, Jaïs P, Haïssaguerre M, Clementy J, Jimenez M. Detrimental ventricular remodeling in patients with congenital complete heart block and chronic right ventricular apical pacing. *Circulation* 2004;**110:**3766-72

23. Andersen HR, Nielsen JC, Thomsen PE, Thuesen L, Mortensen PT, Vesterlund T, Pedersen AK. Long-term follow-up of patients from a randomised trial of atrial versus ventricular pacing for sick-sinus syndrome. Lancet 1997;**350:**1210-6

24. Sweeney MO, Hellkamp AS, Ellenbogen KA, Greenspon AJ, Freedman RA, Lee KL, Lamas GA, for the MOde Selection Trial (MOST) Investigators. Adverse Effect of Ventricular Pacing on Heart Failure and Atrial Fibrillation Among Patients With Normal Baseline QRS Duration in a Clinical Trial of Pacemaker Therapy for Sinus Node Dysfunction *Circulation* 2003;**107:**2932-7

25. Zhang XH, Chen H, Siu CW, Yiu KH, Chan WS, Lee KL, Chan HW, Lee SW, Fu GS, Lau CP, Tse HF. New-onset heart failure after permanent right ventricular apical pacing in patients with acquired high-grade atrioventricular block and normal left ventricular function. *J Cardiovasc Electrophysiol* 2008;**19:**136-41

26. Riahi S, Nielsen JC, Hjortshøj S, Thomsen PE, Højberg S, Møller M, Dalsgaard D, Nielsen T, Asklund M, Friis EV, Christensen PD, Simonsen EH, Eriksen UH, Jensen GV, Svendsen JH, Toff WD, Healey JS, Andersen HR; DANPACE Investigators. Heart failure in patients with sick sinus syndrome treated with single lead atrial or dual-chamber pacing: no association with pacing mode or right ventricular pacing site. *Europace* 2012;**14:**1475-82

27. Nielsen JC, Kristensen L, Andersen HR, Mortensen PT, Pedersen OL, Pedersen AK. A randomized comparison of atrial and dual-chamber pacing in 177 consecutive patients with sick sinus syndrome: echocardiographic and clinical outcome. *J Am Coll Cardiol* 2003;42:614-23

28. Ahmed M, Gorcsan J 3rd, Marek J, Ryo K, Haugaa K, R Ludwig D, Schwartzman D. Right ventricular apical pacing-induced left ventricular dyssynchrony is associated with a subsequent decline in ejection fraction. *Heart Rhythm* 2014;**11:**602-8

29. Pap R, Gallardo R, Rónaszéki D, Agoston G, Traykov VB, Sághy L, Varga A, Forster T. The role of pacing-induced dyssynchrony in left ventricular remodeling associated with long-term right ventricular pacing for atrioventricular block. *J Electrocardiol* 2012;45:357-60

30. Wilkoff BL, Cook JR, Epstein AE, Greene HL, Hallstrom AP, Hsia H, Kutalek SP, Sharma A; Dual Chamber and VVI Implantable Defibrillator Trial Investigators. Dual-chamber pacing or ventricular backup pacing in patients with an implantable defibrillator: the Dual Chamber and VVI Implantable Defibrillator (DAVID) Trial. *JAMA* 2002;**288:**3115-23

31. Brunner M, Olschewski M, Geibel A, Bode C, Zehender M. Long-term survival after pacemaker implantation. Prognostic importance of gender and baseline patient characteristics. *Eur Heart J* 2004;**25:**88-95

32. Jahangir A, Shen WK, Neubauer SA, Ballard DJ, Hammill SC, Hodge DO, Lohse CM, Gersh BJ, Hayes DL. Relation between mode of pacing and long-term survival in the very elderly. *J Am Coll Cardiol* 1999;**33:**1208-16

33. Shen WK, Hammill SC, Hayes DL, Packer DL, Bailey KR, Ballard DJ, Gersh BJ. Long-term survival after pacemaker implantation for heart block in patients > or = 65 years. *Am J Cardiol* 1994;**74:**560-4

34. Shen WK, Hayes DL, Hammill SC, Bailey KR, Ballard DJ, Gersh BJ. Survival and functional independence after implantation of a permanent pacemaker in octogenarians and nonagenarians. A population-based study. *Ann Intern Med* 1996;**125:**476-80

35. Jelić V, Belkić K, Djordjević M, Kocović D. Survival in 1,431 pacemaker patients: prognostic factors and comparison with the general population. *PACE* 1992;**15:**141-7

36. Mayosi BM, Little F, Millar RN. Long-term survival after permanent pacemaker implantation in young adults: 30 year experience. *PACE* 1999;**22:**407-12

37. Sweeney MO, Hellkamp AS, Ellenbogen KA, Lamas GA. Reduced ejection fraction, sudden cardiac death, and heart failure death in the mode selection trial (MOST): implications for device selection in elderly patients with sinus node disease. *J Cardiovasc Electrophysiol* 2008;**19:**1160-6

38. Shukla HH, Hellkamp AS, James EA, Flaker GC, Lee KL, Sweeney MO, Lamas GA; Mode Selection Trial (MOST) Investigators. Heart failure hospitalization is more common in pacemaker patients with sinus node dysfunction and a prolonged paced QRS duration. *Heart Rhythm* 2005;**2:**245-51

39. Sweeney MO, Hellkamp AS, Ellenbogen KA, Greenspon AJ, Freedman RA, Lee KL, Lamas GA, for the MOde Selection Trial (MOST) Investigators. Adverse Effect of Ventricular Pacing on Heart Failure and Atrial Fibrillation Among Patients With Normal Baseline QRS Duration in a Clinical Trial of Pacemaker Therapy for Sinus Node Dysfunction *Circulation* 2003;**107:**2932-7

40. Wild DM, Fisher JD, Kim SG, Ferrick KJ, Gross JN, Palma EC. Pacemakers and implantable cardioverter defibrillators: device longevity is more important than smaller size: the patient’s viewpoint. Pacing Clin Electrophysiol2004;27:1526-9

41. Dean J, Sulke N. Pacemaker battery scandal. *Brit Med Journal* 2016;**352;**i228

42. Uslan DZ, Gleva MJ, Warren DK, Mela T, Chung MK, Gottipaty V, Borge R, Dan D, Shinn T, Mitchell K, Holcomb RG, Poole JE. Cardiovascular implantable electronic device replacement infections and prevention: results from the REPLACE registry. *Circulation* 2010;**122:**1553-61

43. Wild DM, Fisher JD, Kim SG, Ferrick KJ, Gross JN, Palma EC. Pacemakers and implantable cardioverter defibrillators: device longevity is more important than smaller size: the patient's viewpoint. *Pacing Clin Electrophysiol* 2004 Nov;27(11):1526-9

44. Rosenthal LS, Mester S, Rakovec P, Penaranda JB, Sherman JR, Sheldon TJ, Zeng C, Wang P; CAPTURE Trial Investigators. Factors influencing pacemaker generator longevity: results from the complete automatic pacing threshold utilization recorded in the CAPTURE Trial. *Pacing Clin Electrophysiol* 2010;**33:**1020-30

45. Boriani G, Rusconi L, Biffi M, Pavia L, Sassara M, Malfitano D, Bongiorni MG, Padeletti L, Filice I, Sanfelici D, Maffei P, Vicentini A, Branzi A. Role of ventricular Autocapture function in increasing longevity of DDDR pacemakers: a prospective study. *Europace* 2006;**8:**216-20

46. Crossley GH, Gayle DD, Simmons TW, Haisty WK, Bailey JR, Davis-O'Brien K, Hammon JW, Fitzgerald DM. Reprogramming pacemakers enhances longevity and is cost-effective. *Circulation* 1996;**94**(9 Suppl):II245-7

47. Stockburger M, Defaye P, Boveda S, Stancak B, Lazarus A, Sipötz J, Nardi S, Rolando M, Moreno J. Safety and efficiency of ventricular pacing prevention with an AAI-DDD changeover mode in patients with sinus node disease or atrioventricular block: impact on battery longevity-a sub-study of the ANSWER trial. *Europace* 2015 Nov 26. pii: euv358. [Epub ahead of print]

**48. Gierula J, Jamil HA, Byrom R, Joy ER, Cubbon RM, Kearney MT, Witte KK. Pacing-associated left ventricular dysfunction? Think reprogramming first! *Heart* 2014;100:765-9**

49 Foley PW, Leyva F, Frenneaux MP. What is treatment success in cardiac resynchronization therapy? *Europace* 2009;**11** Suppl 5:v58-65

# 50. Yu CM, Chau E, Sanderson JE, Fan K, Tang MO, Fung WH, Lin H, Kong SL, Lam YM, Hill MR, Lau CP. Tissue Doppler echocardiographic evidence of reverse remodeling and improved synchronicity by simultaneously delaying regional contraction after biventricular pacing therapy in heart failure. *Circulation* 2002;105:438-45

# 51. Kapetanakis S, Kearney MT, Siva A, Gall N, Cooklin M, Monaghan MJ. Real-time three-dimensional echocardiography: a novel technique to quantify global left ventricular mechanical dyssynchrony. *Circulation* 2005;112:992-1000

# 52. Kapetanakis S, Bhan A, Murgatroyd F, Kearney MT, Gall N, Zhang Q, Yu CM, Monaghan MJ. Real-time 3D echo in patient selection for cardiac resynchronization therapy. *JACC Cardiovasc Imaging* 2011;4:16-26

# 53. Tsang W, Kenny C, Adhya S, Kapetanakis S, Weinert L, Lang RM, Monaghan M. [Interinstitutional measurements of left ventricular volumes, speckle-tracking strain, and dyssynchrony using three-dimensional echocardiography.](http://www.ncbi.nlm.nih.gov/pubmed/24055125) *J Am Soc Echocardiogr* 2013;26:1253-7

# 54. Curtis AB, Adamson PB, Chung E, Sutton MS, Tang F, Worley S. Biventricular versus right ventricular pacing in patients with AV block (BLOCK HF): clinical study design and rationale. *J Cardiovasc Electrophysiol* 2007;18:965-71

# 55. Bossuyt PM, Reitsma JB, Bruns DE, Gatsonis CA, Glasziou PP, Irwig L, LijmerJG Moher D, Rennie D, de Vet HCW, Kressel HY, Rifai N, Golub RM, Altman DG, Hooft L, Korevaar DA, Cohen JF, For the STARD Group. STARD 2015: An Updated List of Essential Items for Reporting Diagnostic Accuracy Studies. *BMJ* 2015;351:h5527

# 56. Collins GS, Reitsma JB, Altman DG, Moons KG. Transparent reporting of a multivariable prediction model for individual prognosis or diagnosis (TRIPOD): The TRIPOD statement. *Circulation* 2015;131:211-9

# 57. Harrell FE. (2006) Regression Modeling Strategies: With Applications to Linear Models, Logistic Regression, and Survival Analysis. Springer, Berlin

58. Hox, J. (2002), Multilevel Analysis. Techniques and Applications, 2nd edition. London, Lawrence Erlbaum

# 59. Konstam MA, [Udelson JE](http://www.ncbi.nlm.nih.gov/pubmed/?term=Udelson%20JE%5BAuthor%5D&cauthor=true&cauthor_uid=14583894), [Anand IS](http://www.ncbi.nlm.nih.gov/pubmed/?term=Anand%20IS%5BAuthor%5D&cauthor=true&cauthor_uid=14583894), Cohn JN. Ventricular remodeling in heart failure: a credible surrogate endpoint. *J Card Fail* 2003;9:350-3

60. Kramer DG, Trikalinos TA, Kent DM, Antonopoulos GV, Konstam MA, Udelson JE. Quantitative evaluation of drug or device effects on ventricular remodeling as predictors of therapeutic effects on mortality in patients with heart failure and reduced ejection fraction: a meta-analytic approach. *J Am Coll Cardiol* 2010;56(5):392-406

61. Yu CM, Bleeker GB, Fung JW, Schalij MJ, Zhang Q, van der Wall EE, Chan YS, Kong SL, Bax JJ. Left ventricular reverse remodeling but not clinical improvement predicts long-term survival after cardiac resynchronization therapy. *Circulation* 2005;**112:**1580-6

62. Wessler BS, Kramer DG, Kelly JL, Trikalinos TA, Kent DM, Konstam MA, Udelson JE. Drug and device effects on peak oxygen consumption, 6-minute walk distance, and natriuretic peptides as predictors of therapeutic effects on mortality in patients with heart failure and reduced ejection fraction. *Circ Heart Fail* 2011;**4:**578-88

63. Gold MR, Daubert C, Abraham WT, Ghio S, St John Sutton M, Hudnall JH, Cerkvenik J, Linde C. The effect of reverse remodeling on long-term survival in mildly symptomatic patients with heart failure receiving cardiac resynchronization therapy: results of the REVERSE study. *Heart Rhythm* 2015;**12:**524-30

64. McMeekin JD, Lautner D, Hanson S et al. Importance of heart rate response during exercise in patients using atrioventricular synchronous and ventricular pacemakers. *Pacing Clin Electrophysiol* 1990;**13:**59-68

65. Batey RL, Sweesy MW, Scala G, Forney RC. Comparison of low rate dual chamber pacing to activity responsive rate variable ventricular pacing. *Pacing Clin Electrophysiol* 1990;**13:**646-52

66. Carmouche DG, Bubien RS, Kay GN. The effect of maximum heart rate on oxygen kinetics and exercise performance at low and high workloads. *Pacing Clin Electrophysiol* 1998;**21**(4 Pt 1):679-86

67. Capucci A, Boriani G, Specchia S, Marinelli M, Santarelli A, Magnani B. Evaluation by cardiopulmonary exercise test of DDDR versus DDD pacing. *Pacing Clin Electrophysiol* 1992;**15**(11 Pt 2):1908-13

68. Lau CP, Rushby J, Leigh-Jones M, Tam CY, Poloniecki J, Ingram A, Sutton R, Camm AJ. Symptomatology and quality of life in patients with rate-responsive pacemakers: a double-blind, randomized, crossover study. *Clin Cardiol* 1989;**12:**505-12

69. Trappe HJ, Klein H, Frank G, Lichtlen PR. Rate-responsive pacing as compared to fixed-rate VVI pacing in patients after ablation of the atrioventricular conduction system. *Eur Heart J* 1988;**9:**642-8

70. Haywood GA, Katritsis D, Ward J, Leigh-Jones M, Ward DE, Camm AJ. Atrial adaptive rate pacing in sick sinus syndrome: effects on exercise capacity and arrhythmias. *Br Heart J* 1993;**69:**174-8

**71. Jamil HA, Gierula J, Paton MF, Byrom R, Lowry JE, Cubbon RM, Cairns DA, Kearney MT, Witte KK. Chronotropic incompetence does not limit exercise capacity in chronic heart failure. *J Am Coll Cardiol* 2016;67:1885-96**
